# The miR-193a-5p/NCX2/AKT axis promotes invasion and metastasis of osteosarcoma

**DOI:** 10.7150/jca.60969

**Published:** 2021-08-08

**Authors:** Ruiqi Chen, Yichong Ning, Guirong Zeng, Hao Zhou, Lin Zhou, Pei Xiao, Zhihong Li, Jianlin Zhou

**Affiliations:** 1 Department of Orthopedics, The Second Xiangya Hospital, Central South University, Changsha, Hunan 410011, China.; 2 Hunan Key Laboratory of Tumor Models and Individualized Medicine, The Second Xiangya Hospital, Central South University, Changsha, Hunan 410011, China.; 3 Key Laboratory of Protein Chemistry and Developmental Biology of the Ministry of Education, College of Life Science, Hunan Normal University, Changsha 410081, Hunan, China.; 4 Hunan Key Laboratory of Pharmacodynamics and Safety Evaluation of New Drugs & Hunan Provincial Research Center for Safety Evaluation of Drugs, Changsha 410331, Hunan, China.

**Keywords:** miR-193a-5p, NCX2, AKT, osteosarcoma, metastasis

## Abstract

MiR-193a-5p has been observed to have oncogenic or tumor suppressive functions in different kinds of cancers, but its role and molecular mechanism in osteosarcoma are elusive. Na^+^/Ca^2+^ exchangers (NCX1, NCX2 and NCX3) normally extrude Ca^2+^ from the cell, and deregulation of the intracellular Ca^2+^ homeostasis is related to several kinds of diseases, including cancer. The present study demonstrated that miR-193a-5p was upregulated in osteosarcoma tissues compared with the corresponding adjacent noncancerous tissues, and promoted colony formation, migration, invasion and epithelial-mesenchymal transition (EMT) in osteosarcoma cells (SaOS-2 and U-2OS), as well as metastasis in a murine xenograft model. Tandem mass tag-based quantitative proteomics analysis identified NCX2 as a potential target of miR-193a-5p. Luciferase activity assays and Western blotting further confirmed that miR-193a-5p recognized the 3′-untranslated region of NCX2 mRNA, and negatively regulated NCX2 expression. NCX2 was downregulated in osteosarcoma tissues, and its expression was negatively correlated with miR-193a-5p levels. Ectopic expression of NCX2 in osteosarcoma cells could reverse the oncogenicity of miR-193a-5p, indicating that miR-193a-5p exerted its effects by targeting NCX2. Further study demonstrated that NCX2 suppresses Ca^2+^-dependent Akt phosphorylation by decreasing intracellular Ca^2+^ concentration, and then inhibited EMT process. Treatment with the antagomir against miR-193a-5p sensitized osteosarcoma to the Akt inhibitor afuresertib in a murine xenograft model. In conclusion, a miR-193a-5p/NCX2/AKT signaling axis contributes to the progression of osteosarcoma, which may provide a new therapeutic target for osteosarcoma treatment.

## Introduction

Osteosarcoma is the most prevalent malignant bone tumor. It commonly occurs in adolescents and young adults, the highest incidence is among those aged 10-19 years 1. The tumors mainly develop in the bones around the knee, either in the distal femur or the proximal tibia 2. Osteosarcoma is a highly aggressive cancer; that is prone to metastasis or recurrence. Approximately 16.7% of patients present lung metastases at initial diagnosis 3, and 30% to 40% of patients have recurrence 4. Although the introduction of adjuvant chemotherapy following surgery has improved the 10-year overall survival (OS) rate from 30% to approximately 50%, no improvement in OS has been achieved since the 1990s 4. Thus, it is necessary to investigate the molecular pathogenesis of osteosarcoma to improve the efficacy of diagnosis and treatment.

MicroRNAs (miRNAs) are a class of short single-stranded RNAs that are involved in various physiological and pathological processes, including cancer progression. MiR-193a-5p plays different roles in different types of cancer. It is aberrantly overexpressed in lung, prostate, pancreas and liver cancers 5-8 and can facilitate migration, invasion and chemoresistance 5, 8-10. Conversely, miR-193a-5p plays a tumor-suppressive role and is downregulated in breast cancer, stomach and oesophageal squamous cell carcinoma 11-13. The role of miR-193a-5p in osteosarcoma is not completely understood. The expression of miR-193a-5p in osteosarcoma clinical samples has not been investigated, and the only two investigations about the role of miR-193a-5p in osteosarcoma were performed *in vitro* with contradictory results 14, 15. Jacques et al showed that miR-193a-5p contributes to cisplatin resistance through inhibition of TAp73β in primary osteosarcoma 14, but Pu et al reported that both miR-193a-5p and miR-193a-3p suppress the migration and invasion of the osteosarcoma cell line MG63 15.

Na^+^/Ca^2+^ exchangers (NCXs), also called solute carrier family 8 (SLC8), are a type of ion transporters that operate by a stoichiometry of three Na^+^ ions for one Ca^2+^ 16, 17. NCXs normally extrude Ca^2+^ from the cell, but also bring Ca^2+^ into the cell under special conditions 16, 17. In mammals, NCXs consist of three members: NCX1 (SLC8A1) has a ubiquitous distribution, while NCX2 (SLC8A2) and NCX3 (SLC8A3) are exclusively expressed in the brain 16, 17. Recently, NCX2 was also found to be expressed in stomach 18 and kidney 19. NCX2 expression is upregulated during RANKL-induced osteoclast differentiation 20. All three NCX proteins play important roles in regulating intracellular Ca^2+^ homeostasis, and deregulation of Ca^2+^ homeostasis is related to several kinds of disease, including cancer 21. Increasing evidence shows that NCXs are involved in tumorigenesis and progression 22-24. It has been reported that NCX2 is silenced by DNA methylation in human glioma 25, and ectopic expression of NCX2 inhibits growth, angiogenesis and invasion of glioblastoma 24. Reactivation of the tumor suppressor Maspin significantly upregulates NCX2 expression in MDA-MB-231 breast cancer cells, suggesting the potential role of NCX2 in breast cancer 26. However, the role of NCX2 in osteosarcoma has not been investigated.

In this study, we demonstrate that miR-193a-5p is upregulated in osteosarcoma and promotes colony formation, migration, invasion, and epithelial-mesenchymal transition (EMT) *in vitro*, as well as metastasis *in vivo*. NCX2 is a direct target of miR-193a-5p, and it suppresses the EMT process by inactivating Ca^2+^-dependent Akt activation. Overall, we identified a novel miR-193a/NCX2/AKT signalling axis in osteosarcoma.

## Materials and methods

### Tissue specimens

Twenty-five pairs of osteosarcoma and matched adjacent noncancerous tissues were collected from the Second Xiangya Hospital, Central South University. The criteria for study entry included: newly diagnosed, no history of other tumors, no other underlying diseases that possibly affect the treatment process, and no special preoperative treatment. The samples were histologically confirmed by haematoxylin and eosin (HE) staining. The clinicopathological characteristics are listed in Supplementary Table S1. This study was approved by the Ethics Committee of the Second Xiangya Hospital.

### Chemicals and antibodies

Chemicals used in the study include afuresertib, SC-79 (Selleck, Houston, TX, USA), ionomycin (Yeasen, Shanghai, China) and Fluo-8 (Sunnyvale, CA, USA). Antibodies used in the study are NCX2 (Invitrogen, Carlsbad, CA, USA), CDH1 (Promab, Changsha, USA), Slug, Snail, AKT1, phospho-AKT1 (S473), HLA-B, ACTB, and GAPDH (ABclonal, Wuhan, China). Antagomir-193a-5p was purchased from RIBOBIO (Guangzhou, China).

### Cell culture

The cell lines SaOS-2, U-2OS, SJSA-1, MG63 and hFOB1.19 were obtained from ATCC (Manassas, VA, USA) and cultured according to the instructions of ATCC. All the cell lines were mycoplasma-free and authenticated using short tandem repeat analysis by Yubo Biological Technology Co., Ltd. (Shanghai, China).

### Lentivirus and transduction

Lentiviruses expressing miR-193a precursor and the miR-193a-5p target sequence (TCATCTCGCCCGCAAAGACCCA) were used to overexpress and interfere with the expression of miR-193a-5p, respectively. A lentivirus expressing a nontarget sequence (TTCTCCGAACGTGTCACGT) was used as a control. All lentiviruses were purchased from GeneChem (Shanghai, China). Infection and selection for stable cell lines were performed as described previously 10.

### Adenovirus and transduction

The adenoviruses expressing NCX2, empty vector, NCX2 shRNA (target sequence: GCACCTGAGAGTCTTCTTT) or scramble shRNA (nontarget sequence: TTCTCCGAACGTGTCACGT) were obtained from Obio Technology Corp (Shanghai, China). Transductions were performed according to the instruction of manufacturers.

### RNA preparation, reverse transcription and qRT-PCR

Total RNA was extracted from cell lines or human osteosarcoma tissues using TRIzol reagent (Invitrogen, Carlsbad, CA, USA). For analysis of mRNA levels, total RNA was reverse-transcribed into cDNA using the All-in-One First-Strand cDNA Synthesis Kit (GeneCopoeia, Guanzhou, China), the cDNA was then used as a template for real-time PCR with the SYBR Green master kit (GeneCopoeia). The primers for NCX2 are AAGCACCTGAGAGTCTTCTTTGT (forward) and, CAGGCGAATACCACGCACA (reverse). The primers for control 18 S rRNA are CGGCGACGACCCATTCGAAC (forward) and GAATCGAACCCTGATTCCCCGTC (reverse). The expression of mature miRNA was detected with an All-in-One miRNA qRT-PCR kit (GeneCopoeia). Briefly, total RNA was first polyadenylated by poly(A) polymerase and converted into cDNA by reverse transcriptase with oligo-dT primer. Then the cDNA was used as a template for real-time PCR with universal primer and miR-193a-5p-specific primer provided in the kit.

### *In vitro* assay

Colony formation assays, wound healing assays and transwell invasion assays were performed as described previously 27.

### Plasmids, transfection and luciferase assay

The wild-type or mutated target sequence (miRNA response element, MRE) of miR-193a-5p were cloned into the dual-luciferase miRNA target expression vector pmirGLO (Promega Corporation, Madison, WI, USA) as described previously 10. The resulting plasmids were co-transfected with miR-193a-5p mimics or scramble miRNA into Hek293 cell. At 24 h post-transfection, luciferase assay was performed as described previously 10.

### Metastatic xenograft model

BALB/c nude male mice (4-6-weeks-old) were purchased from SLACCAS Jingda (Changsha, China). SaOS-2 or U2-OS cells stably overexpressing expressing miR-193a precursor, miR-193a-5p target sequence or nontarget sequence were implanted into nude mice by tail vein injection. After the experiment, the mice were sacrificed, the tumor nodules formed on the lung and liver surfaces were counted, and the lungs and livers were embedded in paraffin for HE staining and immunohistochemistry (IHC) assays.

### Subcutaneous xenograft model

SaOS-2 cells were subcutaneously injected into BALB/c nude male mice (4-6-weeks-old). At 12 days after injection, the mice were randomly divided into 4 groups (5 mice per group), and treated with vehicle + antagomir-scramble, vehicle + antagomir-193a-5p, afuresertib + antagomir-scramble or afuresertib + antagomir-193a-5p. The vehicle and afuresertib (1 mg/mouse) were administered via oral gavage every other day for 16 days. Antagomir-scramble and antagomir-193a-5p (15 μg/mouse) were administered by intratumoral injection every four days for 16 days. The body weight and tumor size were measured every 2 days. After the experiment, the mice were dissected and the tumor was removed and subjected to weighing, imaging and IHC.

### Measure of intracellular calcium concentration

Cells were incubated with Fluo-8 (4 μM) in a 37 °C, 5% CO_2_ incubator for 1 hour, then washed with PBS three times. Then, fluorescence excitation at 492 nm was measured by flow cytometry.

### TMT-labeling LC-MS/MS

The U-2OS cells stably expressing miR-193a precursor, miR-193a-5p target or a non-target sequence were lysed and digested as described by Wisniewski et al. 28. 100 μg peptide mixture of each sample was labeled with 10-plex TMT reagents (Thermo Fisher Scientific). The labeled samples were equally mixed, then fractionated into 8 fractions by Pierce high pH reversed-phase fractionation kit (Thermo fisher scientific). Each fraction was analyzed by nanoLC-MS/MS on a Q Exactive mass spectrometer (Thermo Scientific) that was coupled to Easy nLC (Thermo fisher scientific). MS/MS spectra were searched using MASCOT engine (Matrix Science, London, UK; version 2.2) embedded into Proteome Discoverer 1.4. The mass spectrometry proteomics data have been deposited to the ProteomeXchange Consortium via the PRIDE 29 partner repository with the dataset identifier PXD013987.

### Statistical analysis

All statistical analyses were conducted with SPSS 22.0 (SPSS Inc., Chicago, IL). The values are presented as means ± SD. Differences between two groups were analyzed using Student's t-test, and the frequencies of two groups were compared using the chi-squared test.

## Results

### MiR-193a-5p is upregulated in osteosarcoma

**Figure 1:**
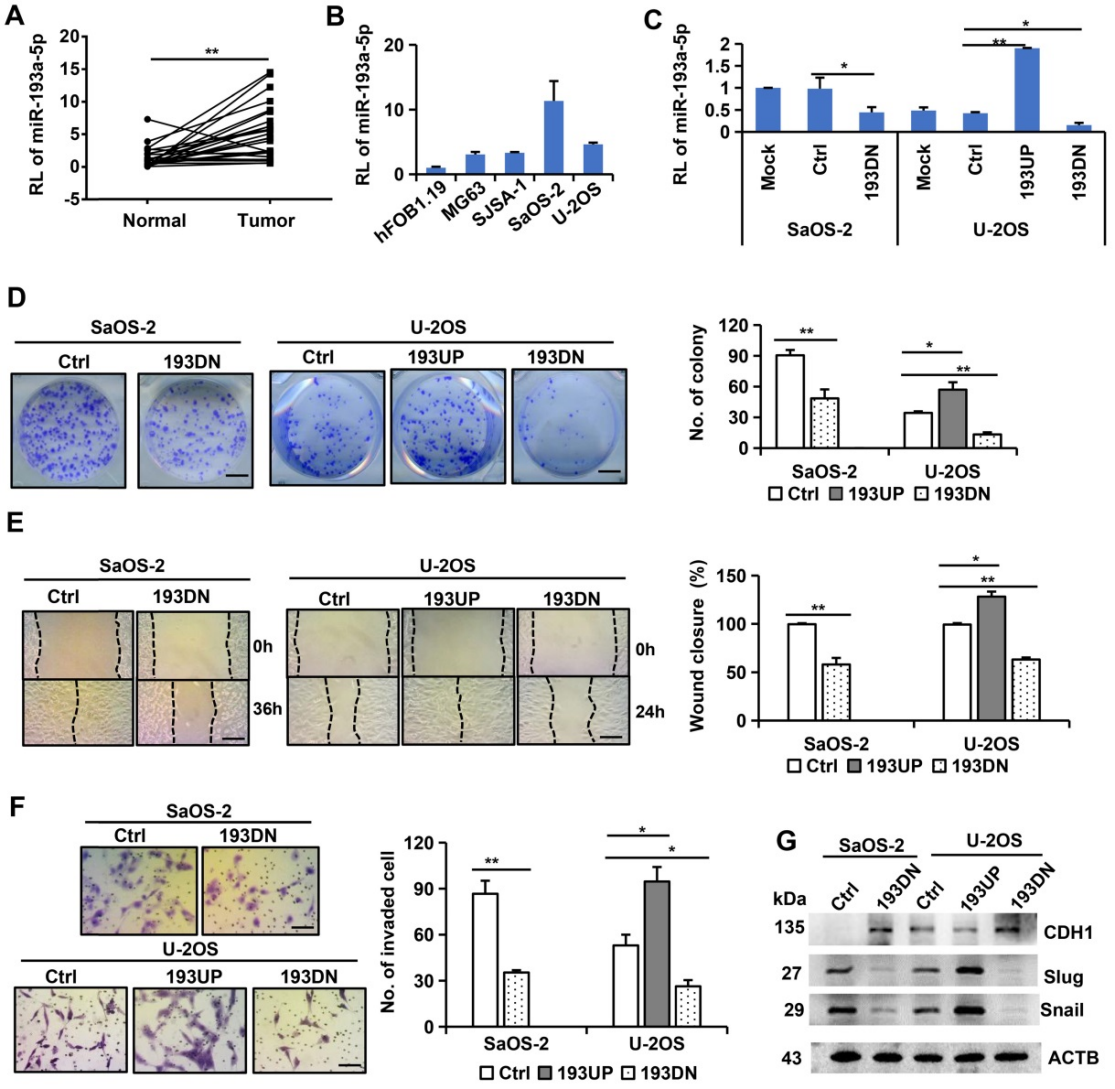
** miR-193a-5p is upregulated in osteosarcoma tissues and promotes colony formation, migration, invasion and EMT. A-C.** RT-qPCR was used to detect relative level (RL) of miR-193a-5p in osteosarcoma tissues (A), cell lines (B) and the cells (C) stably expressing pre-miR-193a (193UP), anti-miR-193a-5p (193DN) and non-target sequence (Ctrl). **D.** Representative images of colony (left panel) and the colony numbers (right panel). Bar length: 1 cm. **E.** Representative images (left panel) of wound area at the indicated time and percentage of wound closure (right panel). Bar length: 20 µm. **F.** Representative images (left panel) and numbers (right panel) of invaded cells in the Transwell invasion assay. Bar length: 20 µm. **G.** The influence of miR-193a-5p on EMT-related marker was determined by Western blotting. All the values were presented as means + SD for three independent experiments. Differences between two groups were analyzed by student t-test; * p ≤ 0.05, ** p ≤ 0.01.

The expression of miR-193a-5p was investigated in 25 pairs of primary osteosarcoma and corresponding adjacent noncancerous tissues by qRT-PCR. The results showed that miR-193a-5p was expressed at significantly higher levels in the tumor tissues than in the corresponding adjacent noncancerous tissues (Figure 1A). Moreover, the level of miR-193a-5p in osteosarcoma cell lines (MG63, SJSA-1, SaOS-2 and U-2OS) was significantly higher than that in the normal human osteoblast hFOB1.19 cell line (Figure 1B).

### MiR-193a-5p promotes colony formation, migration, invasion, and EMT of osteosarcoma cells

To explore the role of miR-193a-5p in osteosarcoma, we used a lentivirus-mediated expression system to interfere with miR-193a-5p expression in SaOS-2 cells, which have high levels of endogenous miR-193a-5p, and to overexpress or interfere with miR-193a-5p in U-2OS cells, which have moderate levels of endogenous miR-193a-5p (Figure 1C). Compared with the cells infected with negative control lentivirus, the cells infected with anti-miR-193a-5p lentivirus formed much fewer colonies, while the cells infected with pre-miR-193a-5p had much more colonies (Figure 1D). Wound healing and transwell invasion assays showed that overexpressing miR-193a-5p promoted, but silencing miR-193a-5p decreased, cell migration and invasion (Figure 1E-F).

EMT plays a key role in malignant progression 30. Thus, we investigated the effects of miR-193a-5p on several EMT markers. Overexpression of miR-193a-5p reduced the expression of the epithelial marker CDH1, but augmented the expression of the mesenchymal cell markers Slug and Snail. Conversely, knockdown of miR-193a-5p increased CDH1 expression, but decreased the expression of Slug and Snail (Figure 1G). These results indicate that miR-193a-5p promote EMT process.

### MiR-193a-5p promotes osteosarcoma metastasis in a xenograft mouse model

**Figure 2:**
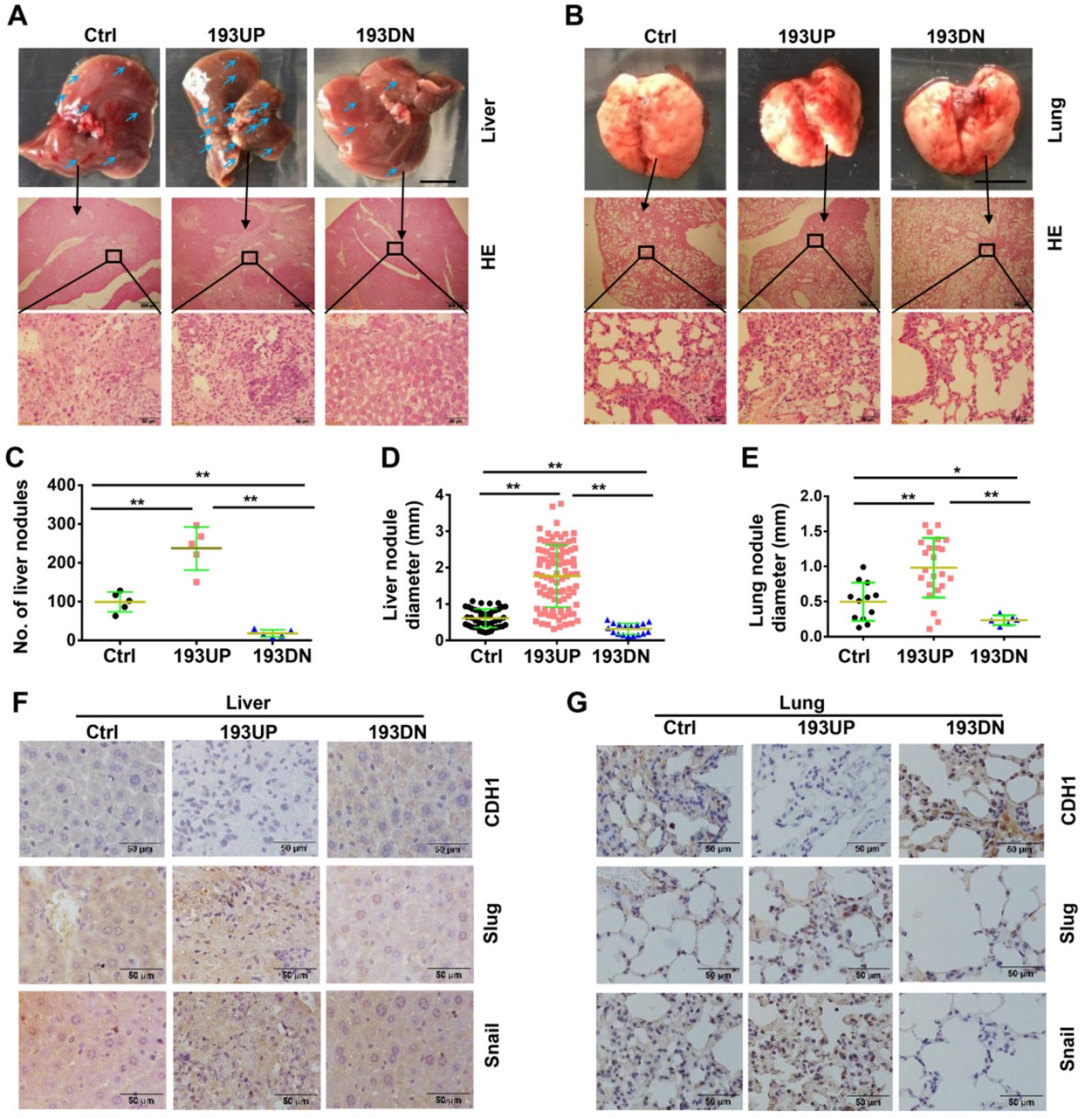
** miR-193a-5p promotes osteosarcoma metastasis to both liver and lung in xenograft mice.** About 1×10^6^ of the U-2OS cells stably over-expressing pre-miR-193a-5p (193UP), ant-miR-193a-5p (193DN) or non-target sequence (Ctrl) were implanted into nude mice (n = 5 per group) by tail-vein injection. At 37 days after injection, the mice were sacrificed. **A-B.** Gross appearance (upper panel, bar length: 1cm) and HE staining (middle panel, bar length: 500 µm; lower panel, bar length: 50 µm) of liver and lung, the arrows indicate the metastatic nodules on the surface of livers. **C.** The number of nodules on liver surface. **D-E.** The diameter of nodules in liver and lung. **F-G.** The expressions of EMT-related markers in liver and lung were determined by IHC. Bar length in the picture of liver and lung: 1cm, bar length in the picture of HE and IHC: 50 µm.

To investigate the effects of miR-193a-5p on metastasis, U-2OS cells stably overexpressing pre-miR-193a-5p, ant-miR-193a-5p or nontarget sequence were implanted into nude mice by tail-vein injection. At 37 days after injection, all mice were euthanized, the metastatic nodules on the surface of the liver and lung were counted, and metastatic lesions were detected by HE staining. Compared with those in mice injected with control cells, the liver metastatic nodules were more and larger in the mice injected with U-2OS-193UP, but fewer and smaller in the mice injected with U-2OS-193DN (Figure 2A, 2C and 2D). There were no visible metastatic nodules on the surface of the lung, but HE staining showed that there were more and larger metastatic nodules in the lung of the pre-miR-193a-group than in the anti-miR-193a-5p group or control group (Figure 2B and 2E). IHC staining showed that miR-193a-5p overexpression decreased CDH1 expression but increased the expression of Snail and Slug, whereas miR-193a-5p knockdown had completely opposite effects in both the liver and lung (Figure 2F-G).

Additionally, we constructed a xenograft mouse model using SaOS-2 cells stably overexpressing pre-miR-193a-5p, ant-miR-193a-5p or nontarget sequence and obtained results (Supplementary Figure S1) consistent with those observed in the U-2OS-derived xenograft mouse model.

### Quantitative proteomics analysis identifies NCX2 as a potential target of miR-193a-5p

**Figure 3:**
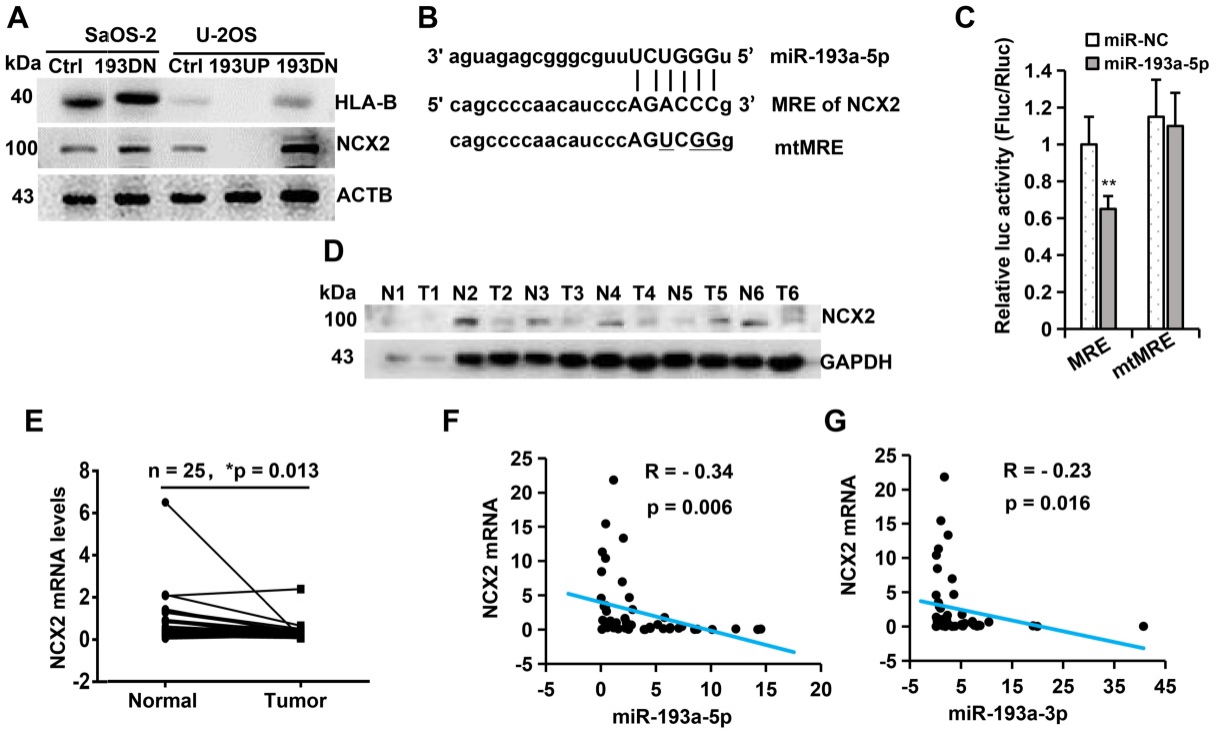
** miR-193a-5p negatively regulates the expression of NCX2 gene. A.** Western blotting was performed to detect the influence of miR-193a-5p on the expression of HLA-B and NCX2. **B.** Diagram of the binding between miR-193a-5p seed sequence and the 3′-UTR of NCX2. The mutated nucleotides in the pmirGLO-mtMRE construct were underlined. **C**. Each luciferase construct (pmirGLO-MRE, pmirGLO-mtMRE) was co-transfected with miR-193a-5p mimics or negative control (miR-NC) into HEK293 cells. At 24 h post-transfection, the luciferase activity was examined and normalized to Renilla luciferase activity. **D.** Western blotting was used to detect the expression of NCX2 in 6 pairs of osteosarcoma (T) and the matched adjacent non-cancerous (N) tissues. **E.** RT-qPCR was performed to detect the mRNA level of NCX2 in 25 pairs of osteosarcoma and the matched adjacent non-cancerous tissues. **F-G.** The correlation between NCX2 level and miR-193a-5p (or miR-193a-3p) in osteosarcoma tissues.

To identify the potential target genes of miR-193a-5p, we used TMT-based quantitative proteomics to analyse the differential expression of proteins following overexpression or knockdown of miR-193a-5p in U-2OS. By analyzing the results of three biological triplicates, we found that 90 proteins were downregulated (fold change <0.833, p<0.05) and 140 proteins were upregulated (fold change > 1.20, p<0.05) after ectopic expression of pre-miR-193a (Supplementary Table S2), while 303 proteins were downregulated and 375 proteins were upregulated following knockdown of miR-193a-5p (Supplementary Table S3). Among the differentially regulated proteins, only two proteins (NCX2 and HLA-B) were both downregulated by pre-miR-193a overexpression and upregulated by knockdown of miR-193a-5p, but no protein was both upregulated by pre-miR-193a overexpression and downregulated by knockdown of miR-193a-5p. Western blotting further confirmed that overexpression of miR-193a-5p in U-2OS decreased, while knockdown of miR-193a-5p in U-2OS or SaOS-2 increased, the levels of NCX2 and HLA-B proteins (Figure 3A). These results suggest that NCX2 and HLA-B are potential targets of miR-193a-5p.

### MiR-193a-5p directly targets NCX2 in osteosarcoma

We then search the potential binding site (miRNA response element, MRE) of miR-193a-5p in the NCX2 and HLA-B genes by miRWalker, a comprehensive database for the prediction of MREs 31. An MRE for miR-193a-5p was identified in the 3′- UTR of NCX2 (Figure 3B), but not in the 3′-UTR, 5′-UTR or coding sequence of the HLA-B gene. To confirm whether the predicted MRE in NCX2 could be recognized by miR-193a-5p, the wildtype or mutant MRE was cloned downstream of the firefly luciferase gene in the pmirGLO vector and cotransfected with miR-193a-5p mimics. Luciferase activity assays showed that miR-193a-5 significantly suppressed the luciferase activity of pmirGLO-MRE, but not that of pmirGLO-mtMRE (Figure 3C). These results suggest that NCX2 is a direct target of miR-193a-5p.

We further detected NCX2 expression in osteosarcoma tissues by qRT-PCR and Western blotting and found that both NCX2 mRNA and protein levels were significantly lower in osteosarcoma tissues than in the corresponding normal tissues (Figure 3D-E). Correlation analysis showed that NCX2 mRNA was negatively corelated with the levels of miR-193a-5p and miR-193a-3p (Figure 3F-G). Compared with miR-193a-5p, miR-193a-3p had a weaker association with NCX2, possibly because NCX2 is mainly regulated by pomiR-193a-5p. The above results indicated that miR-193a-5p recognized NCX2 mRNA and negatively regulated the expression of NCX2.

### MiR-193a-5p promotes colony formation, migration, invasion and EMT by targeting NCX2

**Figure 4:**
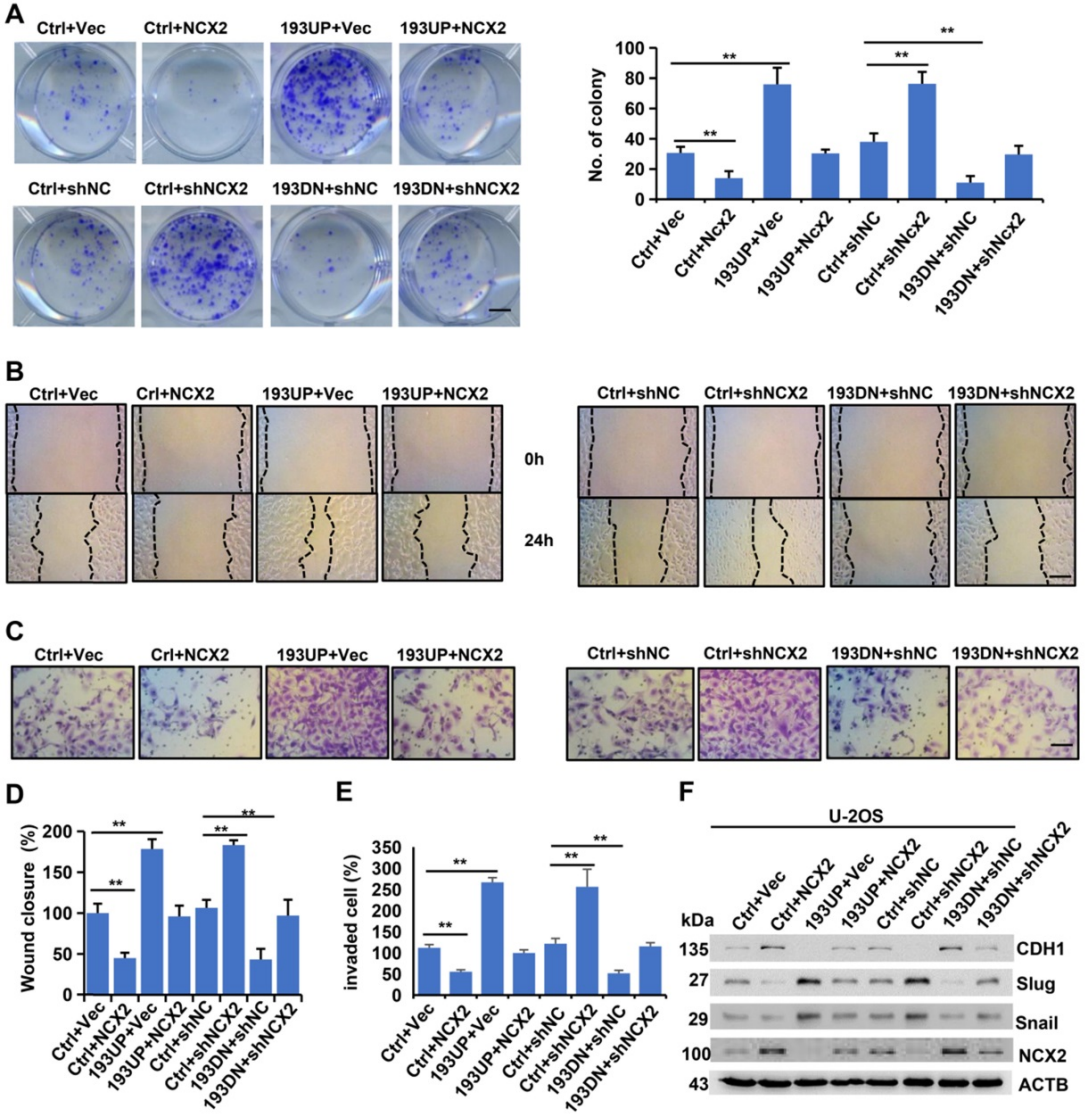
**MiR-193a-5p promotes colony formation, migration, invasion and EMT by targeting NCX2.** The U-2OS cells stably expressing pre-miR-193a (193UP), anti-miR-193a-5p (193DN) or non-target sequence (Ctrl) were infected with the adenovirus expressing NCX2, empty vector (Vec), NCX shRNA (shNCX2), or scramble shRNA (shNC). **A.** Representative images of colony (left panel) and the colony numbers (right panel). **B.** Representative images of wound area at the indicated time. **C.** Representative images of invade cells. **D-E.** Data (mean+SD) represent the result of three independent wound healing assay (**D**) and Transwell invasion assay (**E**). **F.** Western blotting of EMT-related markers. Bar length: 20 µm.

To assess whether miR-193a-5p exerts oncogenic effects by targeting NCX2, we upregulated (or downregulated) NCX2 expression by adenovirus-mediated transfer of its cDNA (or shRNA) into cells and performed *in vitro* assays. As shown in Figure 4A-E, NCX2 overexpression reduced, while miR-193-5p overexpression increased, the colony formation, migration and invasion of U-2OS cells. Moreover, the increase in colony formation, migration and invasion induced by miR-193a-5p overexpression could be abrogated by NCX2 overexpression. Conversely, NCX2 knockdown increased, while miR-193-5p knockdown decreased, colony formation, cell migration and invasion, and the effects of miR-193a-5p knockdown were counteracted by NCX2 knockdown (Figure 4). Additionally, the effects of NCX2 and miR-193a-5p on EMT-related proteins were investigated. Consistent with the above results (Figure 1G), miR-193a-5p overexpression promoted the EMT process, while NCX2 overexpression reduced the effects of miR-193a-5p overexpression on the expressions of EMT-related marker (Figure 4F). Moreover, the effects of miR-193a-5p knockdown were rescued by knockdown of NCX2 (Figure 4F). We repeated the above experiments in SaOS-2 cells and obtained similar results (Supplementary Figure S2). These results suggest miR-193a-5p plays oncogenic roles by targeting NCX2.

### NCX2 suppresses the EMT process by inhibiting Ca^2+^-dependent Akt phosphorylation

**Figure 5:**
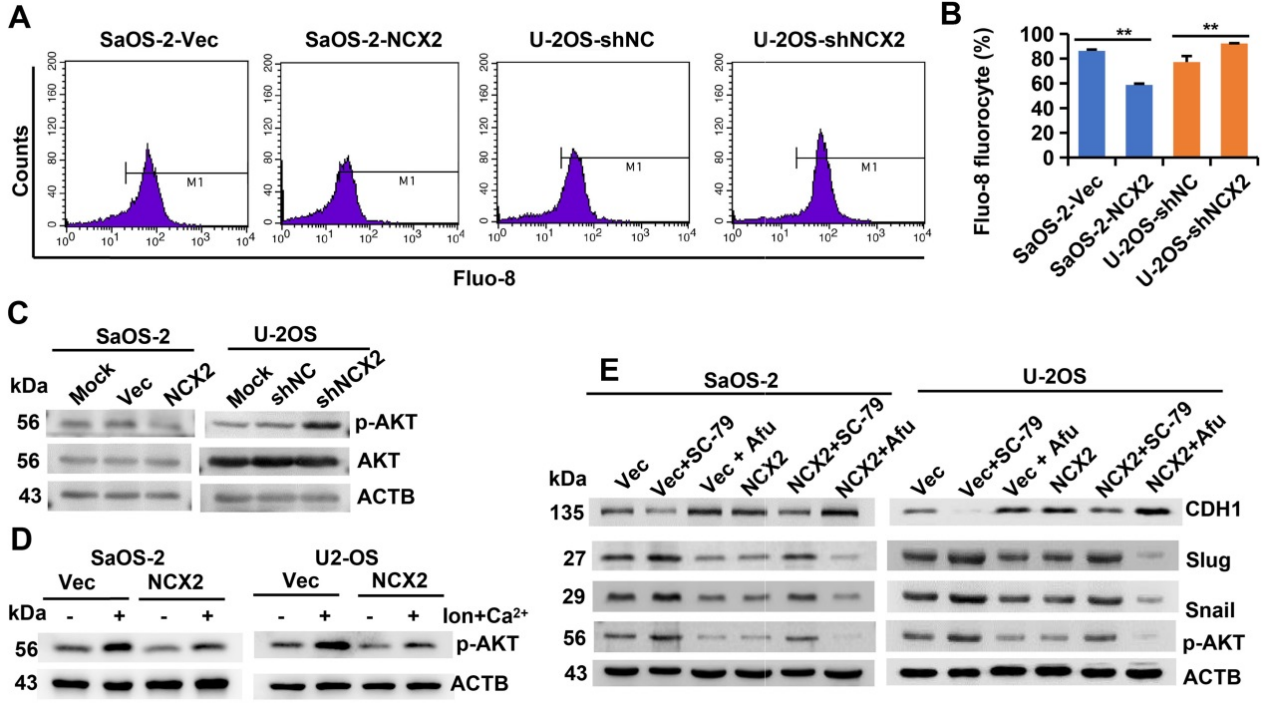
** NCX2 suppresses EMT process by inhibiting Ca^2+^**-**dependent Akt phosphorylation. A-B.** Flow cytometric analysis of intracellular Ca^2+^ concentration after staining with Fluo-8. Representative images (**A**) and corresponding statistical plots (**B**) were shown, respectively. All the values were presented as means + SD for three independent experiments. **C.** Western blotting analysis of phosphorylated and total AKT levels 72 h after infected with adenovirus. **D.** Western blotting analysis of phosphorylated AKT levels. At 72 h after adenovirus introduction, cells were treated with/without ionomycin + Ca^2+^. **E.** Western blotting analysis of EMT-related proteins. Cells were treated with adenovirus, SC-79, or afuresertib (Afu) for 72h, then were subjected for WB.

NCXs normally extrude Ca^2+^ from the cell 16, 17. The Ca^2+^ indicator Fluo-8 was used to measure the influence of NCX2 on intracellular free Ca^2+^ levels. The results showed that overexpression of NCX2 in SaOS-2 cells reduced, while knockdown of NCX2 in U2-OS cells increased, the level of intracellular free Ca^2+^ (Figure 5A-B) indicating that NCX2 promoted Ca^2+^ efflux. It has been reported that AKT is activated by Ca^2+^/calmodulin (CaM)-dependent protein kinase kinase 2 (CaMKK2) in a Ca^2+^/CaM-dependent manner 32. Therefore, we detected the effects of NCX2 on AKT phosphorylation. As expected, NCX2 suppressed the phosphorylation of AKT1 (Figure 5C). Further experiments demonstrated that the suppression of AKT phosphorylation by NCX2 could be rescued by ionomycin-induced Ca^2+^ influx (Figure 5D). These results indicated that NCX2 suppressed AKT phosphorylation by reducing intracellular free Ca^2+^ levels.

Moreover, we investigated whether NCX2 plays a role in the EMT process by affecting AKT phosphorylation. As shown in Figure 5E, the effects of NCX2 overexpression on the expression of EMT-related markers were suppressed by the AKT activator SC-79, but enhanced by the AKT inhibitor afuresertib. The above results suggest that NCX2 suppresses the EMT process by inactivating the Akt pathway.

### Knockdown of miR-193a-5p sensitizes osteosarcoma to afuresertib

**Figure 6:**
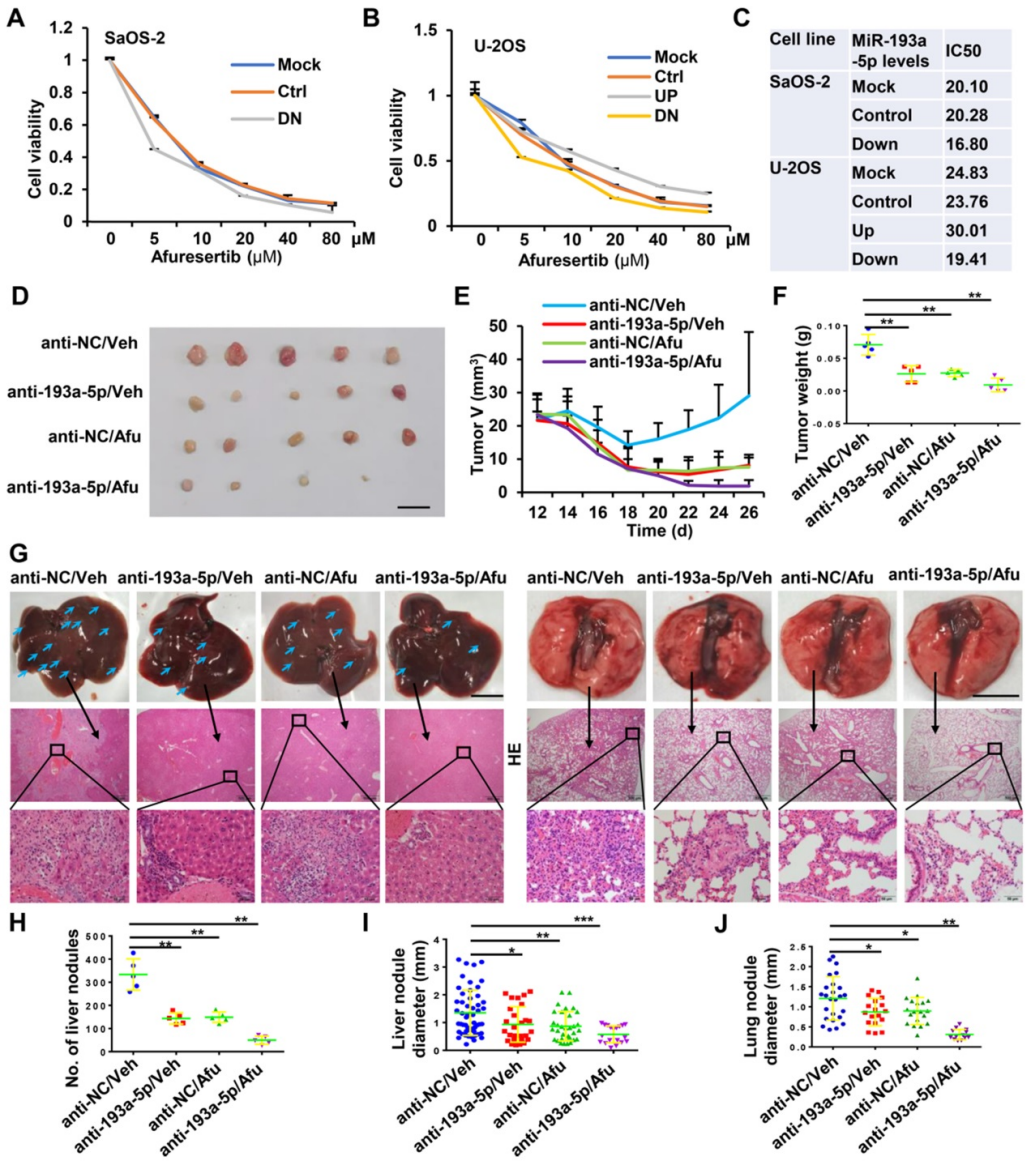
** Knockdown of miR-193a-5p sensitizes osteosarcoma to afuresertib (Afu). A-B.** The influence of miR-193a-5p on Afu cytotoxicity. Cells stably expressing pre-miR-193a (193UP), anti-miR-193a-5p (193DN) or non-target sequence (Ctrl) were treated with the indicated concentration of Afu for 48h, and then cell viability were determined by MTT assy. **C.** The IC50 of Afu. **D-J.** *In vivo* evaluating the anti-tumor efficacy of antagomir-193a-5p (anti-193a-5p) and Afu. Saos-2 cells are subcutaneously injected into BALB/c nude male mice. At 12 days after injection, the mice are randomly divided into 4 groups (5 mice per group), and treated with vehicle (Veh) + antagomir-scramble (anti-NC), Veh + anti-193a-5p, Afu + anti-NC or Afu + anti-193a-5p. **D.** Images of xenograft tumors, bar length: 1 cm. **E.** Dynamic volume of xenograft tumors at different time after injection. **F.** Weight of xenograft tumors. **G.** Gross appearance (upper panel, bar length: 1cm) and HE staining (middle panel, bar length: 500 µm; lower panel, bar length: 50 µm) of liver and lung, the arrows indicate the metastatic nodules on the surface of livers. **H.** number of nodules on liver surface. **I-J.** The diameter of nodules in liver and lung.

Afuresertib is an oral AKT inhibitor with cytotoxic and antiproliferative activities against various kinds of cancer cells. The above results indicated that miR-193a-5p activates AKT by targeting NCX2. Therefore, we explored whether downregulation of miR-193a-5p enhances the effects of afuresertib treatment. *In vitro* assays showed that knockdown of miR-193a-5p decreased, while overexpression of miR-193a-5p increased, the IC50 value of afuresertib in SaOS-2 and U-2OS cells (Figure 6A-C). Then, a subcutaneous tumor model was generated for *in vivo* evaluation of the efficacy of antagomir-193a-5p (a chemically-modified single-stranded miR-193a-5p inhibitor) and afuresertib. The results showed that treatment with antagomir-193a-5p or afuresertib alone could suppress the growth of tumor and metastasis to both liver and lung in osteosarcoma xenograft mice, while the combination treatment had stronger anti-tumor effect (Figure 6D-J).

## Discussion

In this study, we demonstrated that miR-193a-5p was upregulated in osteosarcoma tissues and promoted colony formation, migration, invasion and metastasis. It indicates that miR-193a-5p plays an oncogenic role in osteosarcoma. This result is consistent with the report by Jacques et al 14. Osteosarcoma is prone to lung metastases 3, and the liver is an extremely rare metastatic site of osteosarcoma 33. However, in the metastasis mouse models, we found that miR-193a-5p significantly enhanced osteosarcoma metastasis to both the lung and liver.

Genome-wide miRNA target screening by quantitative proteomics identified NCX2 as a potential target of miR-193a-5p. Luciferase activity assays and Western blotting further confirmed that miR-193a-5p recognized the 3′-UTR of NCX2 mRNA, and negatively suppressed NCX2 expression. In osteosarcoma clinical samples, miR-193a-5p levels were negatively correlated with NCX2 expression. Ectopic expression of NCX2 in osteosarcoma cells could reverse the oncogenic effects of miR-193a-5p. These results indicate that miR-193a-5p exerts its effects by targeting NCX2.

NCXs normally extrude Ca^2+^ from the cell but also bring Ca^2+^ into the cell under special conditions 16, 17. We found that NCX2 levels are negatively correlated with intracellular Ca^2+^ concentrations. Intracellular Ca^2+^ is a second messenger involved in regulating various cellular processes, including cell proliferation and apoptosis in cancer 21, 34. It has been reported that the concentration of intracellular Ca^2+^ is higher in the osteosarcoma cell line MG-63 than the normal osteoblast line hFOB1.19, and activation of calcium-sensing receptor (CaSR) promotes osteosarcoma cell proliferation by upregulating phosphorylation of ERK1/2, PI3K and AKT 35. In ovarian cancer cells, AKT is phosphorylated by CaMKK2 in a Ca^2+^-dependent manner 32. Takahashi et al also demonstrated that TRPA1, a Ca^2+^-influx channel protects cancer cells from apoptotic death by upregulating ERK and PI3K/AKT pathways in a Ca^2+^ -dependent manner 36. These results showed that intracellular Ca^2+^ promoted AKT activation. Our results demonstrated that ectopic expression of NCX2 reduced intracellular Ca^2+^ concentration and AKT phosphorylation, and the effect of NCX2 on AKT phosphorylation could be rescued by increasing Ca^2+^ influx. This indicates that NCX2 possibly prevents AKT activation by decreasing the concentration of intracellular Ca^2+^.

AKT activation has been shown to induce EMT by decreasing CDH1 expression and increasing MMP9 expression 37. Our results showed that NCX2 suppresses EMT by increasing CDH1 expression and decreasing the expression of Slug and Snail. Moreover, the effect of NCX2 on the EMT process could be abrogated by AKT activator, but enhanced by an AKT inhibitor. This indicates that NCX2 suppressed EMT via AKT.

**Figure 7:**
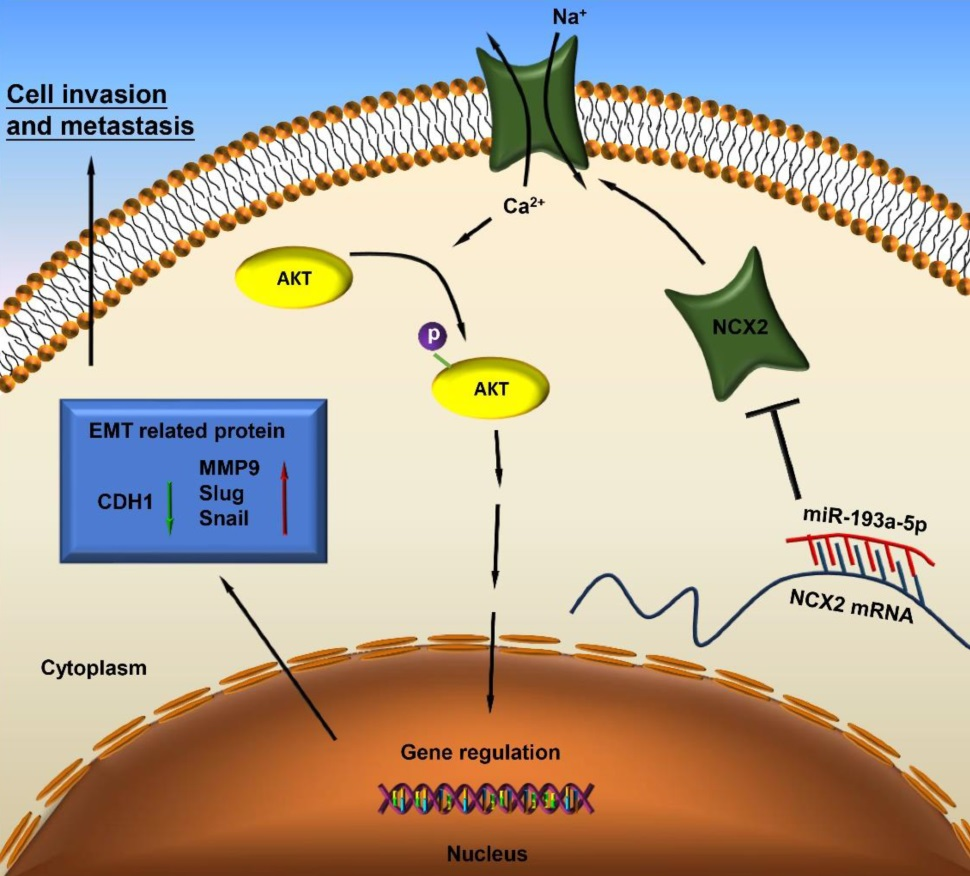
A schematic diagram illustrating the miR-193a-5p/NCX2/AKT axis in osteosarcoma.

Based on our results, miR-193a-5p targets NCX2, while downregulation of NCX2 leads to AKT activation by increasing Ca^2+^ influx, thus facilitating EMT and metastasis of osteosarcoma (Figure 7).

AKT is overactivated in various cancers, and its inhibitors (such as afuresertib and ipatasertib) are promising drug candidates for cancer treatment 38. However, the role of AKT inhibitor for treatment of osteosaroma is not clear. In this paper, afuresertib alone suppressed the growth of tumor in osteosarcoma xenograft mice, while the combination treatment with antagomir-193a-5p had stronger anti-tumor effect. Therefore, antagomir-193a-5p has therapeutic potential in the treatment of osteosarcoma.

In conclusion, we demonstrated that miR-193a-5p was upregulated in osteosarcoma tissues and promoted colony formation, migration, invasion, and EMT *in vitro*, as well as metastasis *in vivo*. Mechanistically, miR-193a-5p targeted NCX2, while NCX2 suppressed the EMT process by inactivating Ca^2+^-dependent AKT activation. Collectively, this study revealed a miR-193a-5p/NCX2/AKT signaling axis in the progression of osteosarcoma, which may provide a new therapeutic target for osteosarcoma treatment.

## Supplementary Material

Supplementary figures.

Supplementary tables.
