# Supplementary material for: The miR-193a-5p/NCX2/AKT axis promotes invasion and metastasis of osteosarcoma
Source: J Cancer. 2021 Aug 8;12(19):5903–13. doi: 10.7150/jca.60969 (PMC8408106; doi:10.7150/jca.60969)
Supplement: Supplementary file 1 — Supplementary figures. [file jcav12p5903s1.pdf]

Supplementary Figure S1 and S2

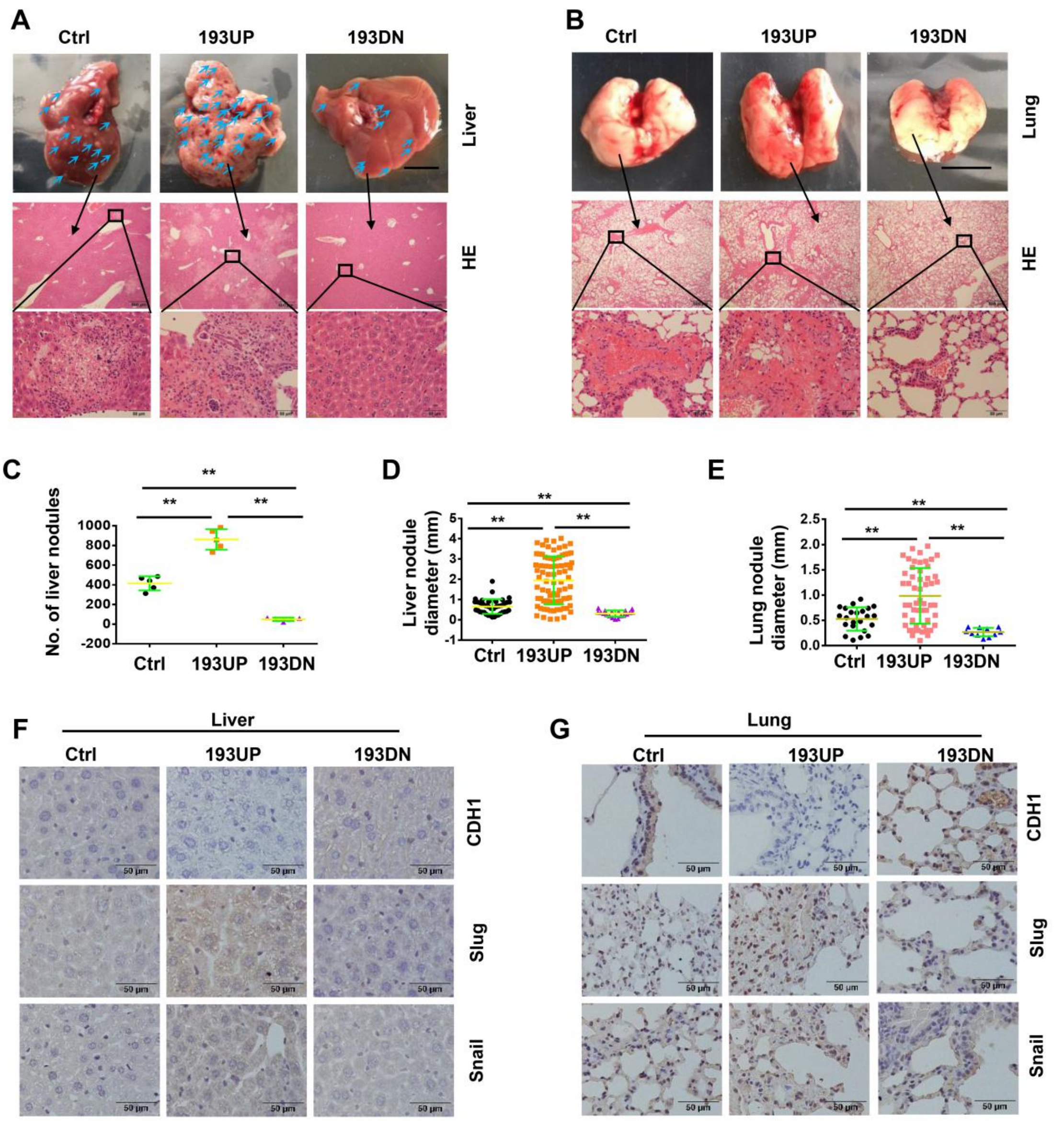

Figure S1. miR-193a-5p promotes osteosarcoma metastasis to both liver and lung in xenograft mice. About 1×106 of the SaOS-2 cells stably over-expressing pre-miR-193a-5p (193UP), ant-miR-193a-5p (193DN) or non-target sequence (Ctrl) were implanted into nude mice (n = 5 per group) by tail-vein injection. At 60 days after injection, the mice were sacrificed. A-B. Gross appearance (upper panel, bar length: 1cm) and HE staining (middle panel, bar length: 500 μm; lower panel, bar length: 50 μm) of liver and lung, the arrows indicate the metastatic nodules on the surface of livers. C. The number of nodules on liver surface. D-E. The diameter of nodules in liver and lung. F-G. The expressions of EMT-related markers in liver and lung were determined by IHC. Bar length in the picture of liver and lung: 1cm, bar length in the picture of HE and IHC: 50 μm.

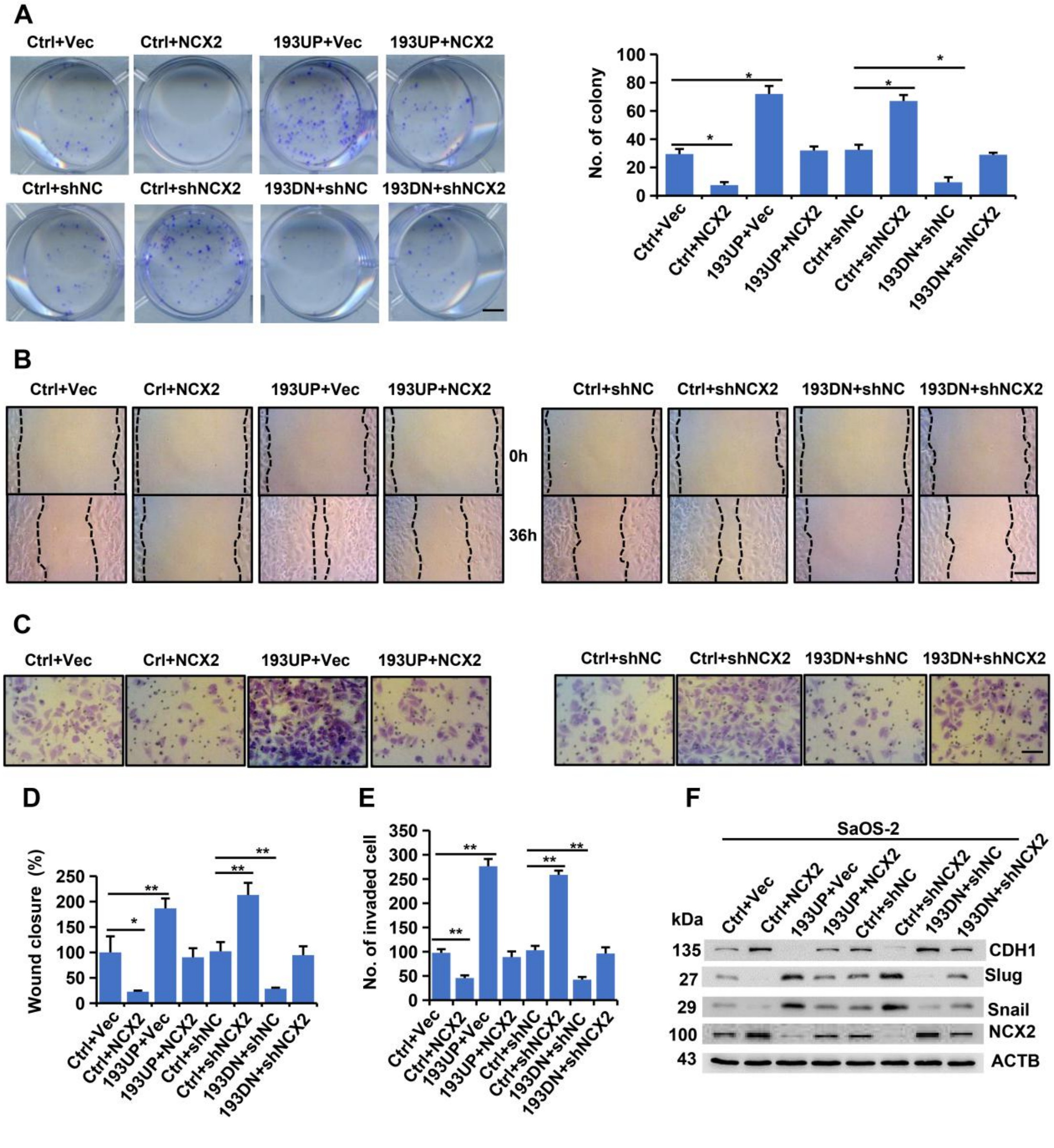

Figure S2. MiR-193a-5p promotes colony formation, migration, invasion and EMT by targeting NCX2. The SaOS-2 cells stably expressing pre-miR-193a (193UP), anti-miR-193a-5p (193DN) or non-target sequence (Ctrl) were infected with the adenovirus expressing NCX2, empty vector (Vec), NCX shRNA (shNCX2), or scramble shRNA (shNC). A. Representative images of colony (left panel) and the colony numbers (right panel). B. Representative images of wound area at the indicated time. C. Representative images of invade cells. D-E. Data (mean+SD) represent the result of three independent wound healing assay and Transwell invasion assay. F. Western blotting of EMT-related markers. Bar length: 20 μm.
